# Targeting deubiquitinase USP28 for cancer therapy

**DOI:** 10.1038/s41419-017-0208-z

**Published:** 2018-02-07

**Authors:** Xiaofang Wang, Zhiyi Liu, Li Zhang, Zhaozhi Yang, Xingxing Chen, Jurui Luo, Zhirui Zhou, Xin Mei, Xiaoli Yu, Zhimin Shao, Yan Feng, Shen Fu, Zhen Zhang, Dongping Wei, Lijun Jia, Jinli Ma, Xiaomao Guo

**Affiliations:** 1 Department of Radiation Oncology, Fudan University Shanghai Cancer Center, Shanghai, 200032 China; 2 Department of Oncology, Shanghai Medical College, Fudan University, Shanghai, 200032 China; 3 Department of Head and Neck Surgery, The University of Texas MD Anderson Cancer Center, Houston, Texas 77030 USA; 4 Department of Breast Surgery, Fudan University Shanghai Cancer Center, Shanghai, 200032 China; 5 Department of Oncology, The First Hospital of Nanjing, Nanjing, 210000 China; 6 Cancer Institute, Longhua Hospital, Shanghai University of Traditional Chinese Medicine, Shanghai, 200032 China

## Abstract

As one of the most important post-translational modifications, ubiquitination plays versatile roles in cancer-related pathways, and is involved in protein metabolism, cell-cycle progression, apoptosis, and transcription. Counteracting the activities of the E3 ligases, the deubiquitylating enzymes have been suggested as another important mechanism to modulate the ubiquitination process, and are implicated in cancer as well. In this article, we review the emerging roles of USP28 in cancer pathways as revealed by recent studies. We discuss the major mechanisms by which USP28 is involved in the cancer-related pathways, whereby USP28 regulates physiological homeostasis of ubiquitination process, DNA-damage response, and cell cycle during genotoxic stress. We further review the studies where USP28 was targeted for treating multiples cancers including non-small cell lung cancer, breast cancer, intestinal cancers, gliomas, and bladder cancer. As a result, the clinical significance of targeting USP28 for cancer therapy merits further exploration and demonstration.

## Facts

- As one of the most prevalent and important post-translational modifications, ubiquitination is involved in multiple cancer-related pathways, including cell-cycle progression, apoptosis, receptor downregulation, and gene transcription.
- Antagonizing the activities of E3 ubiquitin ligases, the deubiquitylating enzymes (DUBs) are engaged in cancer-related pathways by modulating the ubiquitination process.
- The general mode of actions for DUBs in cancer-related pathways are exemplified by their regulation on the stability of oncoproteins or tumor suppressors.
- DUBs have become one of the important cellular targets for cancer treatment.

## Open questions

- Owing to the versatility of USP28-mediated regulation in the cell, what is the dominant mechanism imposed by USP28 for a given cancer type?
- In addition to developing pharmacological inhibitors of USP28, what are other efficient strategies of targeting USP28 that have clinical adaptability and feasibility for treating cancers?

## Introduction

**Fig. 1:**
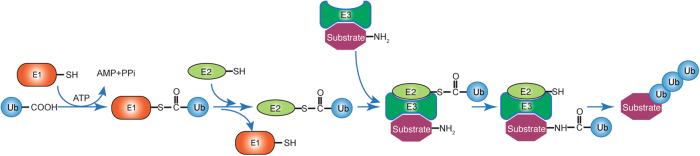
The ubiquitination process. The ubiquitination process is catalyzed by three types of enzymes that function sequentially. The ubiquitin-activating enzyme, E1, promotes the formation of the thioester bond between the C-terminal carboxyl group of ubiquitin and the E1 cysteine sulfhydryl group. This step is dependent on ATP. In the second step, ubiquitin is transferred from E1 to the active site of the conjugating enzyme, E2. In the last step, the E3 ubiquitin ligase catalyzes the attachment of ubiquitin to the substrate through an isopeptide bond between a lysine of the target protein and the glycine of ubiquitin. The E3 enzymes recognize and discriminate various substrates in the cell, thus determining the substrate specificity in a given ubiquitination process. An E3 enzyme typically carries either one of the two domain structures: the homologous to the E6-AP carboxyl terminus (HECT) domain or the really interesting new gene (RING) domain. Depending on the domain it carries, the catalytic mechanisms may differ.

Like phosphorylation, ubiquitination is one of the most prevalent and important post-translational modifications found in the cell, and has multi-faceted roles in normal and pathological cellular processes. In this type of modification, a ubiquitin is attached to the target protein through a covalent isopeptidic bond between the C-terminus of the ubiquitin and a lysine residue of the target protein. Implications of ubiquitination in cancer-related pathways have been unveiled in recent years, and they are mostly related to cell-cycle progression, apoptosis, receptor downregulation, and gene transcription. Three types of enzymes function sequentially to add the ubiquitin to the substrate: the ubiquitin-activating enzyme, E1, catalyzes the formation of a thioester bond on ubiquitin. The E2 ubiquitin-conjugating enzyme carries the ubiquitin. Finally, the E3 ligases attach the ubiquitin from E2 to the substrates (Fig. 1). Ubiquitination was first reported as a mechanism to mark proteins for proteasome-mediated degradation. Later, more functions of ubiquitination have been revealed, spanning from modulating protein trafficking to regulating activities of enzymes^1^.

It has been appreciated that the E3 ubiquitin ligases are central to the ubiquitin-conjugation system since they directly interact with substrates and determine the substrate specificity. Some E3 ligases have been implicated in cancer development because they may dictate degradation of oncoproteins or tumor suppressors. For example, agonists of FBW7 can promote degradation of c-MYC and cyclin E in the tumor cells^2^.

**Fig. 2:**
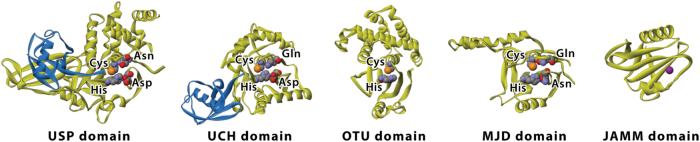
Catalytic domain structures of the five subfamilies of deubiquitinases. The catalytic domain structures of the five subfamilies of deubiquitinases vary significantly. USP, UCH, and MJD catalytic domains share conserved residues around the catalytic core, while OTU domain lacks some of them, and these conserved residues are completely missed in the JAMM domain. (Carbon, gray; nitrogen, blue; oxygen, red; sulfur, orange; zinc, purple. Blue ribbon structures represent the complexed ubiquitin. Reproduced with permission from reference^3^.)

Interestingly, acting reversely by removing the tagged ubiquitin from the substrate protein, deubiquitylating enzymes (DUBs) are implicated as an important mechanism to modulate the ubiquitination process as well, and have been related to cancer, too. There are about 100 DUBs in human, which can be categorized into five families, including ubiquitin-specific proteases (USP), ubiquitin carboxy-terminal hydrolases (UCH), ovarian tumor-like proteases (OTU), metalloproteases, and Machado–Jakob-disease proteases. Except for the DUBs of the JAMM/MPN family, which are metalloproteases, all the other DUBs are cysteine proteases (Fig. 2)^3^. Much knowledge has been accumulated for the functions of DUBs, which are not just limited to reversing ubiquitination of the proteins tagged for proteasomal degradation. It is now known that the functions of DUBs are multi-dimensional, and encompass apoptosis, protein trafficking, cell cycle regulation, DNA damage repair, chromatin remodeling, and modulating signaling pathways^4,5^. Aside from acting on the substrates targeted for degradation, DUBs can also modulate the activities of proteins, therefore affecting the activities of signaling pathways. For instance, in the nuclear factor-κB (NF-κB) pathway, the cylindromatosis-associated DUB, CYLD, negatively regulates NF-κB activation by antagonizing the activity of the E3 ligase tumor necrosis factor receptor-associated factor 2^6–8^. The roles of various DUBs in cancer-associated pathways have been reviewed in^5^. In this review, we focus on discussing the specific roles of USP28 in cancer pathways, and their potential therapeutic values in cancer treatment.

## The roles of USP28 in cancer-related pathways

**Fig. 3:**
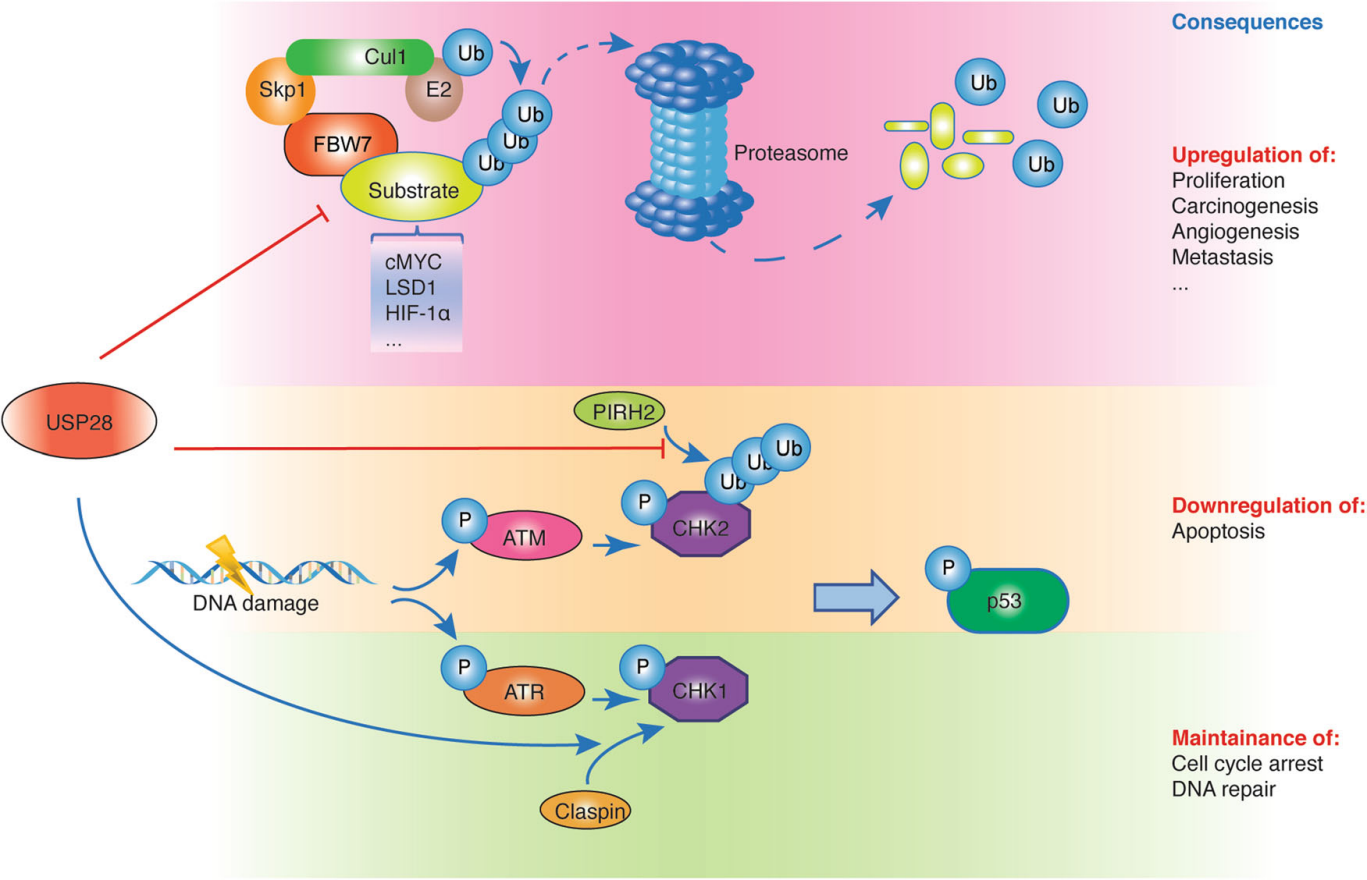
The mechanisms by which USP28 is involved in the cancer-related pathways. (Upper panel) USP28 counteracts the activity of E3 ligases. USP28 antagonizes FBW7, an F-box protein and an important component of the SKP1-CUL1-F-BOX (SCF)-type E3 ubiquitin ligase that targets key transcriptional factors to ubiquitin-directed proteasome degradation. As a result, the stability of some important E3 ligase substrates, such as oncogenic c-MYC, is promoted, leading to enhanced proliferation, carcinogenesis, and metastasis. USP28 also upregulates angiogenesis by antagonizing GSK-3β (glycogen synthase kinase-3β) and FBW7-dependent degradation of HIF-1α (hypoxia-inducible factor-1α), a major regulator of angiogenesis, carcinogenesis, and various processes by which the cell adapts to hypoxic conditions. (Middle panel) USP28 modulates key factors in DNA damage response wherein it forms a complex with PIRH2 and CHK2 and antagonizes PIRH2-mediated poly-ubiquitination and proteasomal degradation of CHK2. Consequently, p53-dependent apoptosis is down-regulated, a process that is favored by the cancer cells. (Lower panel) Genotoxic stress induced by drugs or by ionizing radiation can render the cell to stall before mitosis and repair the resultant DNA damage. USP28 can stabilize Claspin and maintain the G2 arrest, thus promoting the ATR-mediated activation of Chk1. In accordance with its role in checkpoint signaling, Claspin positively regulates DNA replication, and its overexpression can enhance cell proliferation.

USP28 was initially identified through homology search for USP25. Like USP25, the *USP28* gene contains alternatively spliced exons which can produce tissue-specific isoforms of the enzymes^9^. Crystal structures of several USP family enzymes have been resolved, including USP2, USP7, USP8, USP14, and USP21^10–15^. These structural studies suggest that USPs possessing a homologous catalytic site are conferred with a similar ubiquitin-recognition mechanism. Therefore, it is likely that all USP family DUBs may share the same mechanism to recognize ubiquitin. USP28 is highly homologous to USP25, both of which contain ubiquitin-associated domain and ubiquitin-interacting motifs in the N-terminal region^5^. The emerging roles of USP28 in cancer pathways have been revealed by some recent studies. Below, we will review the mechanisms accounting for the involvement of USP28 in these pathways (Fig 3, Table 1), hence demonstrating its significance as a therapeutic target for cancer treatment.

**Table 1:** The reported targets of USP28

USP28 targets	Mode of USP28 action	References
c-MYC	Antagonizes FBW7 activity and stabilizes the FBW7 substrate, c-MYC	^16^
FBW7	Directly stabilizes FBW7 leading to reduction of FBW7 substrates	^18^
c-JUN	Antagonize the ubiquitin-dependent degradation	^18^
Notch1	Antagonize the ubiquitin-dependent degradation	^18^
Claspin	Deubiquitinates and stabilizes the protein	^22^
CHK2	Deubiquitinates and stabilizes the protein	^21,22^
53BP1	Deubiquitinates and stabilizes the protein	^22^
MDC1	Deubiquitinates and stabilizes the protein	^22^
LSD1	Deubiquitinates and stabilizes the protein	^23^
HIF-1α	Antagonizes GSK-3β and FBW7-dependent degradation of HIF-1α	^24^

### Disrupting physiological homeostasis of ubiquitination process

USP28 was the first FBW7-antagonizing DUB in an shRNA screen using c-MYC stability as a readout in human tumor cells^16^. USP28 can counteract the activity of FBW7 and promote c-MYC stability in cancer cells. Overexpression of USP28 inhibited FBW7-depedent ubiquitination of c-MYC and cyclin E1, both of which are oncoproteins. Depletion of USP28 in some cancer cell lines phenocopies the effect of MYC depletion. As a result, USP28 is required for c-MYC-mediated tumor cell proliferation. FBW7 is an F-box protein and an important component of the SKP1-CUL1-F-BOX (SCF)-type E3 ubiquitin ligase, and determines the substrate specificity of the SCF complex. FBW7 is considered as a tumor suppressor that controls cell proliferation, differentiation, and apoptosis by targeting key transcriptional factors to ubiquitin-directed proteasome degradation^17^. Its functions can be counteracted by DUBs, where Lys48-linked ubiquitin chain can be removed by DUBs before the proteolysis of the tagged protein. The functions of DUBs may cover a wide range of physiological processes in the cells; but the most prominent scenario can be exemplified by the promotion of oncoprotein stability. Pathological dysregulation of the deubiquitination process would lead to deleterious effects, causing the cancer-driving oncoproteins to be accumulated.

In addition to stabilizing the FBW7-substrates, USP28 was also found to directly stabilize FBW7 itself, which leads to reduction of FBW7 substrate proteins. As a result, in contrast to the oncogenic effect of overexpressing USP28 in colorectal cancer^18^, inhibition or deletion of USP28 would otherwise have tumor-promoting effect by destabilizing FBW7^19^. This raises an interesting question regarding the dual functions of USP28: whether it is oncogenic or tumor-suppressive? An accountable explanation is that, the levels of USP28 vary considerably in different cells, which can influence the stability and regulation of FBW7 in these cells. As a result, the dual regulation of USP28 on FBW7 may serve as a mechanism to maintain the physiological levels of proto-oncogenic FBW7 substrates. In this model, USP28 may serve as either a tumor promotor or suppressor depending on the status of autocatalytic ubiquitination of FBW7. In the intestine, USP28 deletion does not affect FBW7 stability and attenuates tumorigenesis, while in tissues where FBW7 autocatalytic turnover is favored, deletion of USP28 resembles FBW7 loss-of-function, thus exhibiting a tumor-promoting effect^20^. It is also worth noting that, in addition to the aforementioned oncogenic proteins, other substrates that are known not be associated with FBW7, are also subject to deubiquitination and stabilization by USP28, including Claspin, CHK2, and LSD1^21–23^. This fact adds another layer of complexity regarding the roles of USP28 in cancer-related pathways.

In addition to affecting the turnover of critical oncoproteins like MYC, USP28 may regulate other essential pathways in cancer progression. A study has proposed a novel mechanism by which USP28 upregulates angiogenesis by antagonizing GSK-3β (glycogen synthase kinase-3β) and FBW7-dependent degradation of HIF-1α (hypoxia-inducible factor-1α), a major regulator of angiogenesis, carcinogenesis, and various processes by which cells adapt to hypoxic conditions. HIF-1α is degraded after phosphorylation by GSK-3β and recruitment of FBW7, a process that can be reversed by overexpressing USP28. Importantly, stabilization of HIF-1α by USP28 is dependent on FBW7, and as a result, FBW7 and USP28 reciprocally regulate angiogenesis and metastasis in an HIF-1α-dependent manner^24^. Therefore, modulation of HIF-1 functions by GSK-3/FBW7/USP28 may contribute to a novel adaptive mechanism for the cell to respond to various signals that affect cell proliferation, differentiation, and apoptosis. The finding offers an alternative way to regulate HIF-1–dependent cancer pathways by targeting USP28.

### Tuning DNA-damage response (DDR)

In cancer treatment, radiotherapy complements surgery and chemotherapy. Despite the developments in planning and application of radiotherapy that result in improved cure rate, challenges of radioresistance and radiation-induced damage persist to impair the success of radiotherapy^25–27^. The major effect of radiotherapy is the ionizing damages caused to the DNA, where base damage, single strand break (SSB), and double strand break (DSB) can occur. The cell has exquisite mechanisms to sense and repair the DNA damages. But if these pathways are disturbed and dysregulated, as observed in the tumor cells, radioresistance can develop and nullify the therapeutic efforts. An early study has shown USP28 plays an important role in the control of the DDR^22^. The DDR signals are sensed and initiated by phosphatidylinositol 3-kinase-like kinases (PIKKs), such as ATM and ATR, transduced by various cellular factors, before activating their downstream checkpoint kinases like CHK1 and CHK2. PIKK specificity factors recruit PIKKs to different forms of DNA damage (i.e., base damage, SSB, and DSB) and regulate their activities. Checkpoint mediators, including 53BP1, serve as scaffolds for PIKK complexes and their substrates. Theses checkpoint mediators are critical in controlling the response to irradiation (IR)-induced DDR^28^. USP28 was initially identified as a 53BP1-binding partner in a tandem affinity purification experiment. Mechanistically, USP28 deubiquitinates and stabilizes the specificity factors and mediators of ATM and ATR signaling by protecting them from ubiquitination-mediated proteasome degradation. The regulation on these factors and mediators, including 53BP1, Claspin, and MDC1, is dependent on the physical association with USP28. The catalytic domain of USP28 is required for this regulation as well, and a dominant-negative mutant of a catalytic cysteine phenocopies USP28 knockdown cells. Therefore, USP28 may restrain ubiquitination-mediated proteolysis of the components in DDR. Interestingly, in another study, CHK2 is shown to be directly regulated by USP28. The ubiquitin E3 ligase PIRH2 (p53-induced protein with a RING-H2 domain) interacts with CHK2 and mediates its ubiquitination-directed proteasomal degradation. USP28 forms a complex with PIRH2 and CHK2 and counteracts the negative regulation of PIRH2 on CHK2^21^. Although in this system, the phenotype of USP28-deficient cells is unclear, CHK2 is expected to be destabilized by knockdown of USP28, which may lead to the negative regulation of DDR. However, it is worth noting that regulation of DDR by USP28 has another layer of complexity—USP28 is involved in multiple pathways that can have opposing functions. It can either promote apoptotic death by stabilizing factors of the CHK2-p53 pathway, or promote IR resistance by regulating Claspin^22,29^. Therefore, the effect of USP28 on survival during DDR is determined by the net balance of these opposing pathways in the cell under the physiological milieus, and can be tissue—and cell type-dependent.

### Maintaining G2 arrest during genotoxic stress

During genotoxic stress induced either by drugs or by IR, the cell has to repair the resultant DNA damage before progressing to mitosis. USP28 has been shown to maintain the G2 arrest by stabilizing Claspin, a key regulator of CHK1^30^. Claspin can stimulate ATR-dependent CHK1 activation after genotoxic stress. After the stress is relieved during G2 phase when the cell is recovered from DNA replication stress, Claspin is ubiquitinated and degraded via SCF^βTrcp^. Therefore, degradation of Claspin serves as a mechanism for the cell to shut off the checkpoint and enter the M phase during the cell cycle. Interestingly, Claspin level oscillates between the previous and next rounds of G1/S transition, and thus is subject to active degradation^31–34^. Proteolysis of Claspin in G1 phase is required for the cell to maintain the G0/G1 state and prevent premature entry into S phase. Also, in parallel with its role in checkpoint signaling, Claspin positively regulates DNA replication—overexpression of Claspin can enhance both tumor and normal cell proliferation^29^. As a result, it is likely that stabilization of Claspin by USP28 may have further implications in cell cycle regulation that have not yet been identified. Additionally, in this system, USP28 only targets Claspin but not PLK1 (both of which are subject to ubiquitination by the same E3 ligase). This raises another question—while deubiquitinase activity of USP28 is required to revert ubiquitination-mediated protein turnover, how it discriminates the different substrates remains elusive. Answers to this question can promote our understanding of the pathophysiological implications of USP28-mediated regulation on genotoxicity and cell cycle progression, which may shed light on improving strategies for cancer therapy.

## Targeting USP28 for cancer therapy

DUBs have long been targeted for cancer treatment. For instance, CYLD, a member of the USP class of DUBs, was identified as the causal gene for the rare familial conditions of cylindromatosis and trichoepithelioma, of which the symptom is the development of multiple skin tumors. Loss of function of CYLD tiptoes the transcriptional activity of the NF-κB pathway to the active side and drives many oncogenic processes^6–8,13,35–38^. Therefore, targeting CYLD may have important significance in the treatment of multiple cancers in lung, liver, colon, multiple myeloma, and melanoma^39–42^. Besides CYLD, additional DUBs, including A20, OTUD7B, USP11, H2A, BAP1, USP8, OTUD6B, UCH37, VCPIP1, USP7, COPS5, USP6, USP2, and USP15 have been implicated as potential targets in various cancers^43–54^. The clinical implications of dysregulated USP28 have been documented for various cancers as well. As a result, the significance of targeting USP28 for cancer treatment has been drawn more attention than before. Below, we will review the advances in USP28-targeted cancer therapy (Table 2).

**Table 2:** Molecular mechanisms of USP28-associated pathways in various cancers

Cancer type	Mechanism/pathway	Clinicopathological features	Remarks	References
Non-small cell lung cancer (NSCLC)	USP28 promotes NSCLC cell proliferation and inhibits apoptosis.	Upregulation of USP28 is correlated with poor overall survivals and prognosis in NSCLC patients.	It remains to be determined which microRNA targets USP28 in NSCLC (details in the main text). The exact mechanism by which USP28 promotes NSCLC cell proliferation is undetermined yet.	^55,56^
Breast cancer	USP28 stabilizes LSD1, a critical chromatin modulator that controls cell pluripotency and differentiation, by deubiquitination. USP28 is required for maintaining cancer stem cell (CSC)-like characteristics. Additional mechanism suggests that histone deacetylases 5 (HDAC5) promotes stability of USP28, thus enhancing stability of LSD1.	Overexpression of USP28, HDAC5, and LSD1 is positively correlated in breast tumors.		^23,57^
Intestinal cancers	USP28 promotes deubiquitination of important oncogenic proteins including c-MYC, c-JUN, and NOTCH1, thus stabilizing them in the intestine. This function is independent of the E3 ligase FBW7 in the intestinal crypt stem cells.	USP28 is prevalently overexpressed in human colorectal carcinomas. USP28 also enhances intestinal tumorigenesis.		^2,18^
Gliomas	USP28 stimulates glioma cell proliferation both in vitro and in vivo. USP28-indued c-MYC upregulation may contribute to glioma tumorigenecity.	USP28 level is positively correlated with glioma grade and inversely correlated with patient survivals.	The mechanism remains unclear for the positive regulation of USP28 on c-MYC in human gliomas.	^58^
Bladder cancer	It remains unclear for the role of USP28 in bladder cancer.	Upregulation of USP28 is significantly correlated with bladder cancer grade, staging, and recurrence.	The knowledge of the role of USP28 in bladder cancer is very limited.	^59^

### Targeting USP28 in non-small cell lung cancer (NSCLC)

USP28 has been suggested as a therapeutic target for NSCLC. In one study, USP28 was found to be upregulated in the NSCLC tumors. Importantly, poor survivals were correlated with high levels of USP28 in patients. The cancer-promoting role of USP28 was confirmed by in vitro assays where overexpressing USP28 could enhance NSCLC cell proliferation while downregulating it induced apoptosis. Interestingly, USP28 may be targeted by a microRNA, miR-4295, as USP28 3′UTR-driven luciferase activity was significantly reduced by miR-4295^55^.

In a different study, however, miR-3940-5p was identified to target USP28 and CCND1 (Cyclin D1) in NSCLC. miR-3940-5p level was lower in NSCLC tumors than in matched adjacent normal tissues, and correlated with better clinicopathological features. Coincidently, USP28 was upregulated in NSCLC tumors and associated with poor prognosis of NSCLC patients. Therefore, miR-3940-5p may target CCND1 and USP28 to inhibit NSCLC growth. Indeed, downregulation of miR-3940-5p was concomitant with upregulation of CCND1 and USP28 in NSCLC tissues and cell lines. Importantly, miR-3940-5p suppressed proliferation and promoted apoptosis in NSCLC cells. It was verified that CCND1 and USP28 were direct targets of miR-3940-5p, and the phenotypes of NSCLC cell proliferation and apoptosis imposed by miR-3940-5p could be attenuated by overexpression of CCND1 or USP28. Accordingly, the therapeutic value of targeting USP28 was further demonstrated by in vivo experiments where overexpressing miR-3940-5p inhibited the growth of NSCLC tumors. Therefore, USP28 and CCND1 may be promising diagnostic markers and therapeutic targets for NSCLC^56^.

### Targeting USP28 in breast cancer

One study has revealed a critical mechanism underlying the epigenetic regulation by USP28 in breast cancer. An unbiased siRNA screening against all the human DUBs yielded USP28 as a bona fide deubiquitinase of LSD1 (lysine-specific demethylase 1). USP28 can interact with and stabilize LSD1. In clinical tumor samples, USP28 overexpression is correlated with LSD1 upregulation. Interestingly, LSD1 is a critical epigenetic regulator and controls pluripotency and differentiation by demethylating H3K4me1/2. Disrupting USP28 leads to LSD1 destabilization, which suppresses cancer stem cell-like characteristics in vitro and inhibits tumorigenicity in vivo. Therefore, targeting USP28 provides another treatment approach against breast cancer^23^.

In a follow-up study, it was further confirmed that USP28 could modulate epigenetic events in breast cancer. Both LSD1 and histone deacetylases (HDACs) facilitate breast cancer proliferation, and interestingly, HDAC5 could promote the stability of USP28. Overexpression of USP28 largely reversed HDAC5-knockdown-induced LSD1 protein degradation, suggesting HDAC5 positively regulates LSD1 by stabilizing USP28. Therefore, targeting USP28 may tune down the HDAC5/LSD1-mediated epigenetic process that drives breast cancer development and progression^57^.

### Targeting USP28 in intestinal cancer

As stated previously, USP28 can stabilize FBW7-targeted proteins, exhibiting oncogenic activities. Importantly, USP28 is highly expressed in colon cancers, making it a potential pharmacological target to control c-MYC-driving tumor cells. In a mouse model of colorectal cancer, in contrast to the FBW7-deletion phenotype, intestine-specific deletion of USP28 reduced intestinal tumors. Importantly, in mice carrying the tumors, USP28 deletion reduced tumor size and prolonged lifespan^18^. Interestingly, USP28 can also antagonize the ubiquitin-dependent degradation of two additional oncogenic proteins, c-Jun and Notch1, expanding the substrate list that is shared by both USP28 and FBW7. The therapeutic benefit of USP28 in colon cancer was further demonstrated in another study, where USP28 knockout could increase the life expectancy of animals harboring FBW7-deficient colon tumors. Consistently, FBW7 substrates, including c-MYC, c-JUN, NICD1, and cyclin E1, which were upregulated in FBW7-deficient mice, were downregulated in the double-knockout mouse^2^.

### Targeting USP28 in gliomas

The significance of USP28 in glioma tumorigenesis has been demonstrated by its positive regulation on MYC, which is dysregulated in the majority of gliomas and difficult to target directly. It was determined that USP28 was upregulated in human gliomas but not in normal brain tissues. The USP28 protein level in human glioma tissues was directly correlated with glioma grade, and was inversely correlated with patient survivals. Ectopic USP28 expression promoted proliferation of SW1783 glioma cells both in vitro and in vivo, and conferred enhanced tumorigenicity in a nude mouse model. Disruption of USP28 in glioblastoma U373 cells suppressed anchorage-independent growth in vitro and tumorigenicity in vivo. Importantly, USP28 promoted MYC expression, which was required for USP28-induced cell growth in human glioma. Therefore, USP28 could be a new target of therapy for human malignant gliomas^58^.

### Targeting USP28 in bladder cancer

The clinical significance of USP28 was also demonstrated in human bladder cancer. It has been determined that USP28 was overexpressed in bladder cancers compared to adjacent non-cancerous tissues at both the mRNA and protein levels. IHC examination confirmed that a majority of cancerous tissues displayed high USP28 levels. Furthermore, USP28 was strongly correlated with histopathological grade, clinical stage, tumor number, and recurrence rate. Interestingly, USP28 expression level can serve as an independent predictor of survival. Therefore, USP28 can potentially be valuable in the prognostic evaluation of bladder cancer^59^.

### Pharmacological studies for DUBs

To date, pharmacological studies of DUBs have been focused on screening and identifying novel DUB inhibitors or antagonists for clinical regiment use. High throughput screenings in chemical libraries followed by structural–activity relationship studies have yielded fruitful compounds exhibiting selectivity for particular DUBs^60–63^. Due to the important role of USP7 in stabilizing p53, small molecules selective for USP7 have been a focus of the pharmacological studies for DUBs^64–67^. HBX 41108 was discovered to inhibit USP7 activity at a submicromolar range. Treatment with this compound leads to stabilization of p53 and subsequent repression of cancer cell growth^66^. A dual inhibitor selective for USP7 and USP47 was also reported to promote p53-induced apoptosis in cancer cells. Its efficacy was documented for a mouse xenograft model of myeloma and B-cell leukemia^64^. In Table 3, we list some major USP inhibitors^68–73^. However, despite the advances in DUB-targeting drug discovery, the identified compounds are still in the early stage awaiting clinical approvals. To date, no compounds targeting USP28 have been reported yet, and no DUB-targeted drugs have yet been approved for clinical use. Structural studies were performed for only a handful of DUBs. Therefore, the challenges will offer new opportunities for the drug discovery of clinically relevant DUBs, including USP28.

**Table 3:** USP inhibitors and their pharmacological modes of action

Inhibitor	DUB targets	Mode of action	References
b-AP15	USP14/UCHL5	Induces apoptosis and cell cycle arrest and triggers endoplasmic reticulum stress response signaling in multiple myeloma cells	^69^
IU1(1-[1-(4-fluorophenyl)-2,5-dimethylpyrrol-3-yl]-2-pyrrolidin-1-ylethanone)	USP14	Enhances proteasome activity through inhibition of USP14	^70^
HBX 41,108	USP7	Stabilizes p53 and induces p53-dependent apoptosis	^66^
HBX 19,818	USP7	Forms stoichiometric complex with USP7 and selectively inhibits USP7	^65^
HBX-28,258	USP7	Selectively inhibits USP7	^65^
P022077	USP7	Selectively inhibits USP7	^71^
9-Oxo-9H-indeno[1,2-b]pyrazine-2,3-dicarbonitrile analogues	USP7/USP8	Introduction of O-alkyloxime moieties confers selectivity for USP8	^72^

## Concluding remarks

Ubiquitination is one of the important post-translational modifications observed in the cell and plays multifaceted roles in cellular pathways, affecting protein turnover, signaling, and response to various stresses. DUBs, by removing the ubiquitin tags, have been shown to regulate stability of signaling proteins, which in turn affect the outputs of a variety of signaling cascades controlling cell proliferation, survival, DNA damage response, carcinogenesis, and metastasis. Several DUBs have been targeted for therapeutic applications, as exemplified by some promising drug discovery studies. To date, major roles of USP28 are demonstrated by its regulations of DDR and ubiquitination-dependent proteolysis pathways. It is likely that the functions of USP28 are cell/tissue type-dependent, and in a particular system, a specific aspect of these functions can be dominant. Regardless, USP28 may be a potentially beneficial therapeutic target for cancer treatment. Hence, its clinical relatedness and applications should be extensively explored, investigated, and evaluated.
